# Safety Profile and Neurocognitive Function Following Acute 4-Fluoroamphetamine (4-FA) Administration in Humans

**DOI:** 10.3389/fphar.2018.00713

**Published:** 2018-07-06

**Authors:** Elizabeth B. de Sousa Fernandes Perna, Eef L. Theunissen, Patrick C. Dolder, Natasha L. Mason, Nadia R. P. W. Hutten, Stefan W. Toennes, Kim P. C. Kuypers, Johannes G. Ramaekers

**Affiliations:** ^1^Department of Neuropsychology and Psychopharmacology, Faculty of Psychology and Neuroscience, Maastricht University, Maastricht, Netherlands; ^2^Department of Forensic Toxicology, Institute of Legal Medicine, Goethe University Frankfurt, Frankfurt, Germany

**Keywords:** 4-FA, phase-1, safety, neurocognition, novel psychoactive substance

## Abstract

Availability of novel psychoactive substances (NPS) exponentially increased over the last years. Risk evaluations of NPS are hampered by the lack of pharmacological studies in humans on health parameters. The aim of the present study was to evaluate safety and neurocognitive function of healthy volunteers (*N* = 12) who received single doses of 100 and 150 mg 4-fluoroamphetamine (4-FA), a phenethylamine that has been associated with severe cardiovascular and cerebrovascular complications. The study was set-up as a placebo controlled, within subject, phase 1 trial as it was the first to administer 4-FA to humans under controlled conditions. Overall, 4-FA produced a strong elevation in blood pressure up until 4–5 h after administration that was followed by a sustained increase in heart rate. After an interim review of safety data from five participants, a decision was taken to cancel administration of 150 mg. We subsequently obtained complete datasets for placebo and 100 mg 4-FA treatments only. Effects of 4-FA on mood and neurocognitive function were most distinct at 1 h post drug and included significant elevations of vigor, friendliness, elation, arousal, positive mood, as well as improvements in attention and motor performance. Negative affect was also reported as time progressed in the acute phase and even more so during the subacute phase. Overall, the influence of 4-FA on vital signs, mood, and neurocognition was similar to that observed with other stimulants. Present findings confirm clinical observations of acute toxicity among 4-FA users and warrant warnings about potential health risks associated with 4-FA use.

## Introduction

The global drug market has undergone significant changes over the past years, with novel psychoactive substances (NPS) emerging at a fast pace (EMCDDA, 2017; Logan et al., 2017; Tracy et al., 2017). NPS are defined as novel substances which are not controlled by the United Nations’ 1961 Narcotic Drugs or the 1971 Psychotropic Substances Conventions. A large number of NPS belong to the broad class of phenethylamines that includes typical psychostimulant drugs like amphetamine, methamphetamine, and MDMA (“ecstasy”). A phenethylamine that gained particular popularity in the Netherlands and other EU countries is 4-fluoroamphetamine (4-FA), also known as 4-FMP, 4-Flava, or Flux (Hondebrink et al., 2015). 4-FA has been on the EU drug market since 2007 and its use substantially increased over the years (Nugteren-van Lonkhuyzen et al., 2015; Wijers et al., 2017). Users perceive 4-FA as a “lighter version” of MDMA that produces positive “euphoric” effects, while limiting negative effects such as confusion and dizziness, and has been established as a drug of choice in the last few years (Linsen et al., 2015).

Psychoactive effects of phenethylamines depend on the release of monoamines and the inhibition of their reuptake (Marona-Lewicka et al., 1995; Baumann et al., 2012; Hondebrink et al., 2018). In animals, 4-FA was a more potent releaser of serotonin (5-HT) compared to amphetamine (Wee et al., 2005; Baumann et al., 2011) and increased extra-cellular dopamine levels but with less potency than amphetamine (Marona-Lewicka et al., 1995). Overall, however, the effects of 4-FA on the dopaminergic system are stronger than those on the serotonergic system. Administration of 4-FA to rats increased dopamine levels by 300% while serotonin levels only increased by 30% (Baumann et al., 2011).

Clinical signs of 4-FA use are in line with this pharmacological profile and include sympathomimetic effects such as decreased appetite, sweating, coordination difficulties, mydriasis, insomnia, increased energy, agitation, tachycardia, hypertension, and hyperthermia as well as subjective effects such as increased happiness, euphoria, and empathy (Nugteren-van Lonkhuyzen et al., 2015). Users with 4-FA intoxications often complain of headaches and cardiovascular and cerebrovascular complications such as intracerebral hemorrhages (Wijers et al., 2017).

Scientific data on the behavioral effects of 4-FA in humans is scarce. The current study aimed to fill this gap and was the first to determine the safety and behavioral profile of 4-FA in a placebo-controlled phase 1 trial following escalating doses of 100 and 150 mg, which are the most common doses among 4-FA consumers (Linsen et al., 2015). Participants were kept under close medical supervision for up to 12 h after drug intake to monitor their health and wellbeing. During this period, vital signs, subjective experience, mood, and neurocognitive functions were recorded. It was expected that 4-FA would produce behavioral effects that have previously been reported with MDMA and amphetamine.

## Methods

### Participants

Twelve participants (five females, seven males) entered the study. Their age ranged between 19 and 31 years [mean (*SD*): 22.3 (3.4)]. Their weight ranged between 52 and 88 kg [mean (*SD*): 70.8 (11.6)]. All participants had previous experience with stimulant drugs such as MDMA (11 participants), amphetamine (9 participants), cocaine (6 participants), and 4-FA (5 participants). History of cannabis use and psychedelics was reported by seven and four participants, respectively. All participants consumed alcohol. Participants were recruited through advertisements placed in Maastricht University buildings and by word-of-mouth. All participants provided written informed consent and underwent a medical examination including physical examination, routine laboratory tests (e.g., clinical chemistry, hematology, serology, and urinalyses), vital signs, and electrocardiogram (ECG). Inclusion criteria included: (a) age 18–40 years, (b) the absence of any major medical, endocrine, and neurological condition, (c) free from psychotropic medication, (d) good physical health, (e) body mass index within 18.5–28 kg/m^2^, and (f) experience with psychostimulants (once a week at most and at least one time during the previous year), as we did not wish to promote psychostimulant use among stimulant naïve users. Exclusion criteria included: (a) addiction, (b) history of psychiatric or neurological disorder, (c) pregnancy, (d) cardiovascular, gastrointestinal, hepatic, or renal abnormalities, (e) excessive smoking (>15 cigarettes per day) or drinking (>20 standard units of alcohol per week), (f) hypertension (diastolic >90 mmHg; systolic >140 mmHg), and (e) being a blood donor.

### Design and Treatments

This study was conducted according to a phase 1, single-blind, placebo-controlled, cross-over design. Participants received single doses of 4-FA (100 and 150 mg) or placebo on separate days. 4-FA was dissolved in approximately 150 ml of bitter lemon (Royal Club) and administered orally. Placebo drinks consisted solely of bitter lemon. An escalating dosing scheme was used. Participants received one of the following treatment orders: 0–100–150, 100–0–150, or 100–150–0 mg. Conditions were separated by a minimum washout period of 7 days to avoid carry-over effects.

An independent Data Safety Monitoring Board (DSMB) was installed to evaluate vital and behavioral data collected throughout the study. The sample was divided into two batches of six participants. The second batch would only start after a positive DSMB evaluation of all adverse events experienced during the first batch. The 150 mg dose condition was only initiated after all participants of the first batch completed the 100 mg dose condition. Interim DSMB evaluations were scheduled after every third and sixth participant that completed the 100 and 150 mg dose.

The study was conducted according to the code of ethics on human experimentation established by the declaration of Helsinki (1964) and its amendments and was approved by the Medical Ethics Committee of Maastricht University. A permit from the Dutch drug enforcement administration was acquired for obtaining, storing, and administering 4-FA. Participants received monetary compensation for their participation in the study. The study was registered in the Dutch Trial Register (trial number: NTR6164).

### Procedure

Participants were asked to refrain from drug use at least 1 week prior to the start and during the study. Participants were not allowed to consume alcohol (48 h), caffeine-containing beverages (24 h), or tobacco before and during experimental sessions and were requested to arrive well rested. Alcohol breathalyzing and urine drug screens were conducted on test days after arrival of the participants. Urine drug screens (Instant-View^®^, Multipanel 10 Test Drug Screen, Alfa Scientific Designs Inc., Poway, CA, United States) checked for the presence of amphetamine, barbiturates, benzodiazepines, cocaine, methamphetamine, morphine, methadone, phencyclidine, tetrahydrocannabinol (THC), and MDMA. Treatments were only administered after negative drug and alcohol screens and (when applicable) negative pregnancy tests. Subjective drug experience and mood, vital signs, and neurocognitive function were assessed repeatedly throughout 12 h after treatment, while under medical supervision. At the end of the test day, participants were given a diary in which they were asked to take note of any possible side effect experienced up until 5 days after drug administration. A schematic representation of data collected on test days is given in **Table 1**. Participants received a training session prior to the experimental sessions in order to familiarize them with the neurocognitive tests and minimize practice effects.

**Table 1:** Schematic representation of activities on a test day (B = baseline).

Events	Time after drug administration (hours)													
	B	0	1	2	3	4	5	6	7	8	9	10	11	12
*Drug screen*	^∗^													
*Treatment administration*		^∗^												
Critical tracking task			^∗^			^∗^				^∗^				
Divided attention task			^∗^			^∗^				^∗^				
Digit symbol substitution task			^∗^			^∗^				^∗^				
Spatial memory task			^∗^											
Tower of London				^∗^						^∗^				
Profile of mood scale	^∗^		^∗^			^∗^							^∗^	
*Lunch/break/dinner*					^∗^			^∗^				^∗^		
Lab safety	^∗^							^∗^						
Vital signs	^∗^	^∗^	^∗^	^∗^	^∗^	^∗^	^∗^	^∗^	^∗^	^∗^	^∗^	^∗^	^∗^	^∗^
Blood sample	^∗^	^∗^	^∗^	^∗^	^∗^	^∗^		^∗^		^∗^		^∗^		^∗^

### Safety Measures

A Dyna-Vision ambulatory patient monitoring system (Techmedic International, Netherlands) was used to continuously monitor ECG and vital signs (i.e., saturation of peripheral oxygen, plethysmogram, respiration and skin temperature, and beat-to-beat non-invasive blood pressure) in real time. Blood and urine samples were taken at baseline, 6 h after drug administration and at the end of a test day for hematology, clinical chemistry, and urinalysis.

### Pharmacokinetics

Blood samples to quantify 4-FA concentrations were collected at baseline and after drug administration. Blood samples were centrifuged immediately and the serum was subsequently frozen at −20°C until analyses for pharmacokinetic assessment. 4-FA serum concentrations were quantified after liquid–liquid extraction of 0.5 ml serum by a validated liquid chromatography-tandem mass spectrometry method (0.04 ng/ml lower limit of quantification).

### Neurocognitive Assessment

The Digit Symbol Substitution Task is a computerized version of the original paper and pencil test taken from the Wechsler Adult Intelligence Scale. The participant is shown an encoding scheme consisting of a row of squares at the top of the screen, wherein nine digits are randomly associated with particular symbols. The task is to match each digit with a symbol from the encoding list and click the corresponding response button. The number of digits correctly encoded within 3 min is the performance measure.

The Tower of London (Shallice, 1982) task consists of computer-generated images of begin- and end-arrangements of three colored balls on three sticks. The participant decides as quickly as possible, whether the end-arrangement can be accomplished in two, three, four, or five steps from the begin arrangement by pushing the corresponding number coded button. The number of correct decisions is the main performance measure.

The Spatial Memory Task (Sambeth et al., 2009) consists of an immediate and a delayed relocation phase. The immediate relocation phase is composed of six trials in which 10 black-and-white pictures (total 60 pictures) are subsequently presented at different locations on a computer screen. The participants have to remember and indicate the location of the pictures. The delayed relocation phase starts 30 min later when the pictures reappear in a random order in the middle of the screen and participants again indicate the correct picture location. Dependent variables are number of correct immediate relocations, mean immediate reaction time, delayed relocation score, and mean delayed reaction time.

The Critical Tracking Task (Jex et al., 1966) measures the participant’s ability to control a displayed error signal in a first-order compensatory tracking task. The participants are required to make compensatory movements with a progressively higher frequency. Eventually the response frequency lags the error signal by 180°. At that point, the participant’s response adds to, rather than subtracts from, the error and control is lost. The frequency at which control loss occurs is referred to as “lambda-c” (the “critical frequency”).

The Divided Attention Task assesses one’s ability to divide attention between two tasks performed simultaneously (Moskowitz, 1973). The primary task requires the use of a joystick to continuously null the horizontal movement of a cursor from the center of a display. Tracking error is measured by the absolute distance (millimeter) between the cursor’s position and the center. As a secondary task, the participant monitors 24 single digits in the corners of the computer screen. The requirement is to react as rapidly as possible by lifting the foot from a pedal any time a target, the target number “2,” appears. Mean tracking error, number of control losses, the number of correct detections, false alarms, and the mean reaction time to targets are the dependent variables.

### Subjective Questionnaires

Subjective drug experience was measured using a 100-mm visual analog scale with “not influenced by 4-FA at all” at one end and “very influenced by 4-FA” on the other end of the line. Mood was assessed with the profile of mood scale (POMS). The POMS is a self-assessment mood questionnaire with 72 items, rated on a five-point Likert scale, with 0 being “not at all” to 4 “extremely.” Eight mood states are classified and quantified by calculating the sum score of associated items for each mood state, i.e., anxiety, depression, anger, vigor, fatigue, confusion, friendliness, and elation. Two composite scales are derived, arousal, and positive mood (de Wit et al., 2002).

## Statistics

The effects of 4-FA on all dependent measures were analyzed by means of a general linear model (GLM) repeated measures ANOVA with main factors treatment, time, and the interaction between treatment × time. POMS parameters were analyzed as change scores from baseline, reducing the number of time points to 3. If the sphericity assumption was violated, the Greenhouse–Geisser correction was used. The alpha criterion significance level was set at *p* = 0.05. All statistical tests were conducted with IBM SPSS version 24.0.

## Results

### DSMB Evaluations

The DSMB recommended interfering with the research protocol on two separate occasions. The first recommendation was to withhold the high-dose condition from a participant that experienced a strong increase in systolic/diastolic blood pressure (150/130 mmHg), and heart rate (±165 bpm) approximately 20–30 min after intake of 100 mg 4-FA. The participant felt light headed and looked pale. Blood pressure and pulse frequency gradually decreased within 3 h, but pulse remained higher than baseline throughout the day. The second recommendation was to expose participant in the second batch only to the low dose (100 mg) and drop the high dose (150 mg) altogether because all participants in the first batch showed strong increments in blood pressure and heart rate after the high dose (see data below).

### Missing Data

Twelve participants completed their placebo and 100 mg 4-FA condition. Only five participants received the 150 mg dose of 4-FA. Datasets collected during placebo and 100 mg 4-FA entered the statistical model. Data collected after the high-dose lacked statistical power due to the low sample size but were still included in data presentations below to provide descriptive statistics of potential 4-FA effects after the high dose.

### Safety Data, Subjective High, and Pharmacokinetics

Laboratory safety analyses (hematology, clinical chemistry, and urinalyses) showed no clinically relevant deviations from the normal ranges.

**FIGURE 1:**
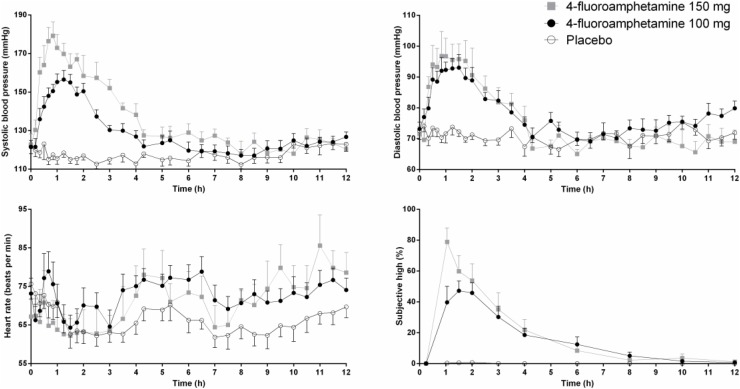
Mean (SE) systolic and diastolic blood pressure, heart rate, and subjective high in every treatment condition as a function of time after treatment.

Mean (SE) systolic and diastolic blood pressure, heart rate, and subjective drug experience following two doses of 4-FA and placebo are presented in **Figure 1**. GLM analyses revealed that 100 mg 4-FA significantly increased systolic blood pressure (*F*_1,11_ = 94,48; *p* = 0.000), diastolic blood pressure (*F*_1,11_ = 31,73; *p* = 0.000), heart rate (*F*_1,11_ = 7,7; *p* = 0.018), and subjective drug experience (*F*_1,11_ = 63,9; *p* = 0.000). 4-FA effects on blood pressure were strongest during 5 h after administration and gradually decreased over time as indicated by a significant treatment × time interaction for systolic (*F*_30,330_ = 12,.78; *p* = 0.000) as well as diastolic measures (*F*_30,330_ = 8,29; *p* = 0.000). The effects of 4-FA on heart rate lasted throughout the entire time window but were most evident between 5 and 12 h as indicated by a significant treatment × time interaction (*F*_30,330_ = 7,7; *p* = 0.018). Subjective high was also elevated throughout the entire window but gradually decreased over time (*F*_9,99_ = 14,35; *p* = 0.000).

**FIGURE 2:**
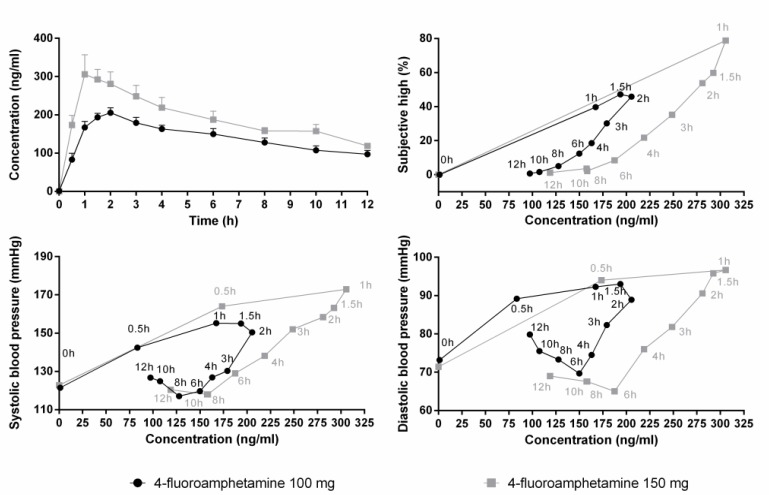
Mean (SE) serum concentrations of 4-FA after 100 (*N* = 12) and 150 mg (*N* = 5) 4-FA (upper panel left). Other panels show that blood pressure and subjective high after both doses of 4-FA follow a clockwise hysteresis when plotted against serum concentrations over time.

Mean (SE) concentrations of 4-FA during the 12-h time window are shown in **Figure 2**. Increments in serum concentrations peaked between 1 and 2 h after administration. 4-FA concentrations slowly decreased over time and but were higher than baseline at 12 h after administration. The influence of 4-FA on subjective high and blood pressure followed a clockwise hysteresis when plotted against serum concentrations over time suggesting the development of acute tolerance, as shown in **Figure 2**.

### Mood

**FIGURE 3:**
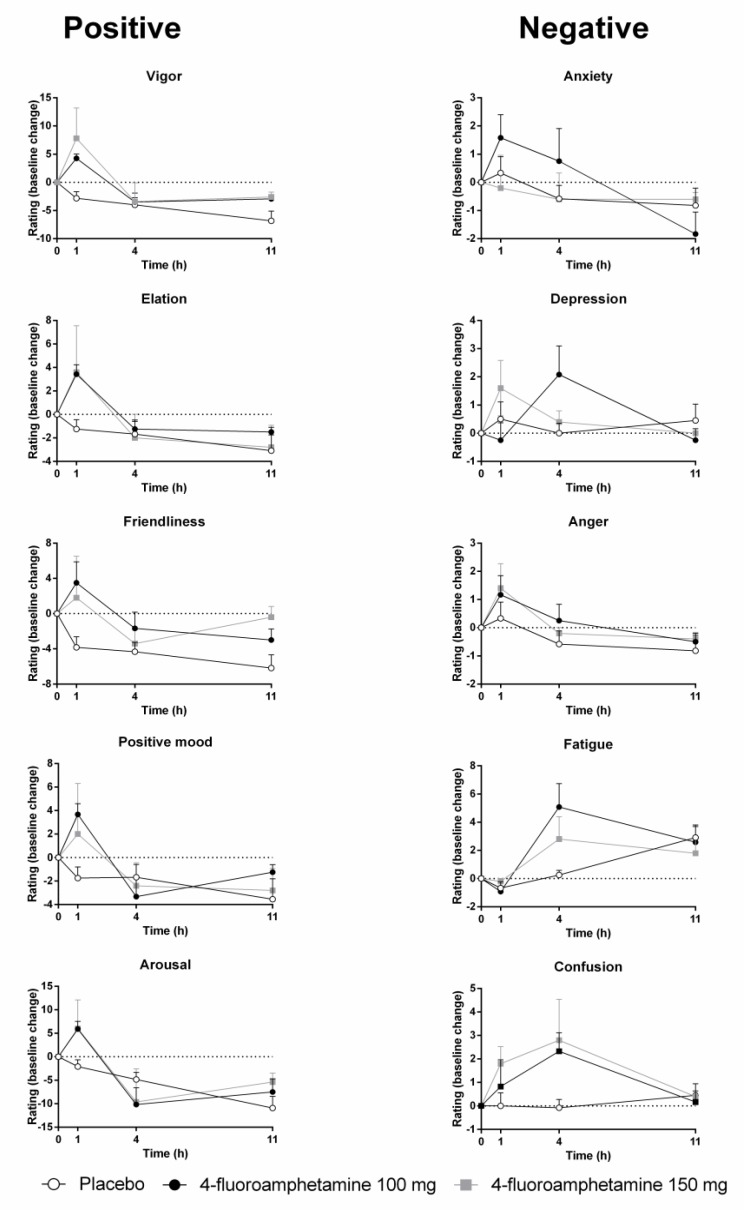
Mean (SE) mood ratings for all POMS items in every treatment condition as a function of time after treatment.

**Figure 3** shows mean subjective ratings of mood as a function of treatment and time after administration. Mood ratings were corrected for baseline. GLM analyses revealed that 4-FA significantly increased ratings of vigor (*F*_1,11_ = 8,43; *p* = 0.002) and friendliness (*F*_1,11_ = 5,55; *p* = 0.038). Treatment × time interactions for vigor (*F*_2,22_ = 10,62; *p* < 0.008) and friendliness (*F*_2,22_ = 4,60; *p* < 0.021) were also significant. Data inspection shows that these ratings were highest at 1 h after administration of 4-FA. Treatment × time interactions also suggested increments in elation (*F*_2,22_ = 4,6; *p* = 0.024), positive mood (*F*_2,22_ = 4,6; *p* = 0.024), anxiety (*F*_2,22_ = 4,41; *p* = 0.025), and arousal (*F*_2,22_ = 4,6; *p* = 0.024), primarily at 1 h after 4-FA. Treatment × time interactions also suggested an increase in fatigue (*F*_2,22_ = 7,51; *p* = 0.003), particularly at 4 h after administration of 4-FA.

### Neurocognitive Performance

**FIGURE 4:**
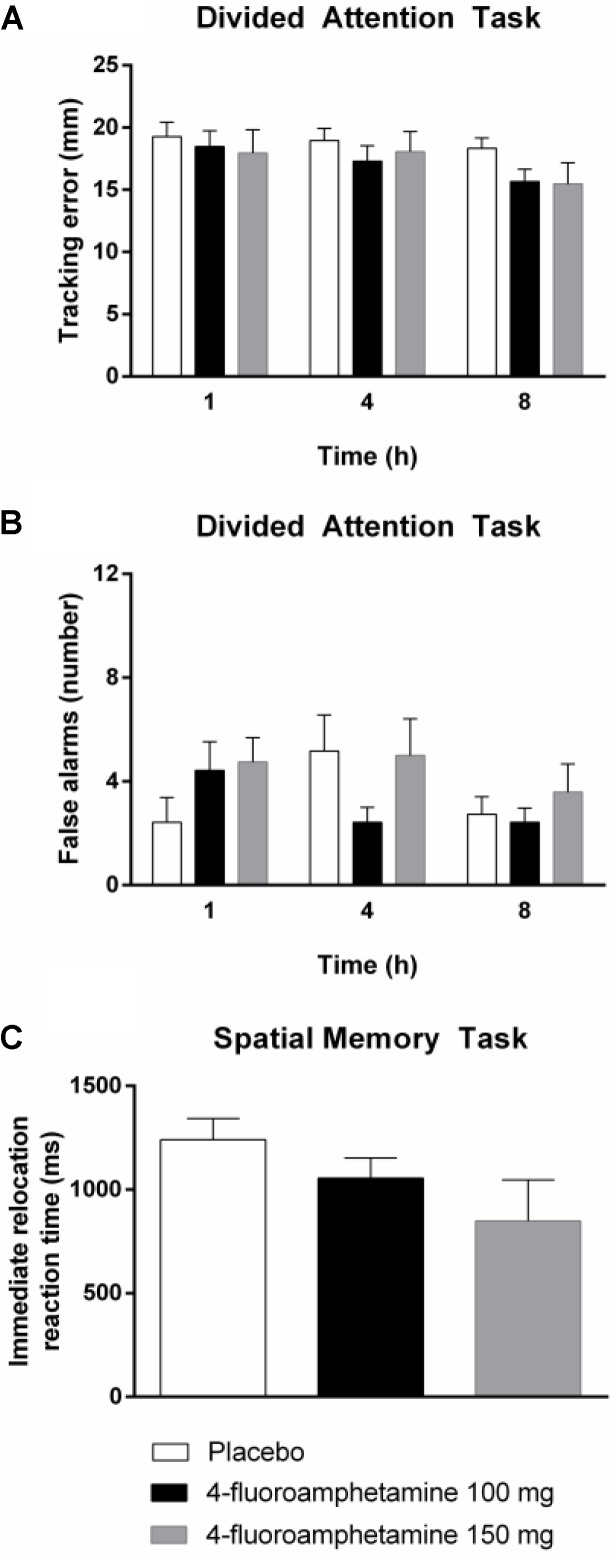
Mean (SE) tracking performance and number of false alarms in the divided attention task **(A,B)** and reaction time in the spatial memory task **(C)** in every treatment condition and as function of time after drug administration.

General linear model analyses revealed that 4-FA decreased immediate relocation reaction time (*F*_1,11_ = 14.40; *p* < 0.003) in the spatial memory task. GLM analyses also revealed that 4-FA reduced tracking error (*F*_1,10_ = 5.49; *p* < 0.041) during the divided attention task. A treatment × time interaction (*F*_2,20_ = 6.25; *p* < 0.008) was observed for false alarms during the divided attention task. Their number was lower following 4-FA, but primarily at 4 h after administration. Mean (SE) values for neurocognitive parameters showing significant treatment effects are shown in **Figure 4**. Mean performance on the immediate relocation score was less following 4-FA but this effect just failed to reach significance (*F*_1,11_ = 3.83; *p* = 0.076). None of the other neurocognitive measures were affected by treatment or treatment × time. Their means are shown in **Table 2**.

**Table 2:** Mean (SE) neurocognitive performance in each treatment condition as a function of time after drug administration.

Neurocognitive parameters	Post drug (hours)	PLA	4-FA, 100 mg	4-FA, 150 mg
**Critical tracking task**				
Lamda-c (rad/s)	1	3.35 (0.18)	3.05 (0.25)	3.77 (0.23)
	4	3.43 (0.20)	3.37 (0.22)	3.47 (0.17)
	8	3.51 (0.23)	3.57 (0.19)	3.93 (0.25)
**Divided attention task**				
Reaction time (s)	1	1.93 (0.10)	1.91 (0.09)	1.76 (0.16)
	4	2.00 (0.82)	1.96 (0.07)	1.59 (0.53)
	8	2.00 (0.12)	1.94 (0.09)	1.70 (0.44)
Correct detections (#)	1	44.33 (4.27)	43.83 (4.40)	43.75 (2.21)
	4	42.00 (7.68)	44.41 (2.57)	40.00 (3.39)
	8	43.09 (5.37)	45.33 (3.05)	44.20(2.33)
Control losses (#)	1	9.41 (3.24)	12.16 (4.06)	12.50 (4.66)
	4	23.08 (7.00)	16.25 (8.04)	33.00 (19.50)
	8	8.63 (3.5)	6.33 (2.31)	9.80 (5.72)
**Digit symbol substitution**				
Number correct (%)	1	92.91 (4.43)	92.25 (3.31)	93.60 (7.15)
	4	91.41 (5.45)	92.25 (3.39)	96.00 (7.43)
	8	94.33 (4.16)	96.66 (3.99)	95.60 (5.94)
**Tower of London**				
Correct decisions (#)	2	32.72 (2.20)	29.70 (2.52)	37.20 (2.47)
	8	38.81 (1.23)	38.81 (.97)	39.0 (1.58)
**Spatial memory task**				
Immediate relocation (#)	1	50.66 (1.06)	47.66 (1.30)	47.40 (1.36)
Delayed relocation (#)	1	43.58 (1.18)	42.91 (1.64)	44.80 (0.86)
Delayed relocation RT (ms)	1	1267 (163)	1.266 (170)	970 (180)

### Adverse Events

A summary of adverse events reported on test days and during the following, sub-acute days is given in **Table 3**.

**Table 3:** Number (percentage) of reported adverse events during 12 h after treatment (acute) and up to 5 d after treatment (subacute).

Adverse events	Acute			Subacute		
	PLA	4-FA, 100 mg	4-FA, 150 mg	PLA	4-FA, 100 mg	4-FA, 150 mg
Headache	1 (8)	1 (8)	1 (8)	3 (25)	4 (33)	1 (20)
Fatigue	–	–	–	1 (8)	5 (42)	2 (40)
Difficulty concentrating	–	–	–	–	3 (25)	1 (20)
Dizziness/light headed	–	4 (33)	–	–	1 (8)	–
Anhedonia	–	–	–	1 (8)	3 (25)	2 (40)
Positive mood	–	–	–	4 (33)	2 (16)	3 (25)
Nausea	–	–	1 (8)	–	–	–
Lack of appetite	–	–	–	–	1 (8)	–
Sleep disturbance	–	–	–	–	2 (24)	–
Irritability	–	–	–	–	2 (24)	–
Muscle tension	–	–	–	–	1 (8)	1 (20)
Perspiration	–	–	–	–	1 (8)	–

## Discussion

The present study aimed to evaluate safety and neurocognitive function of healthy volunteers after receiving single doses of 100 and 150 mg 4-FA. Overall, 4-FA produced significant elevations in heart rate and blood pressure but symptoms of tachycardia and hypertension were most pronounced after the highest dose. The DSMB advised to cancel the highest dose condition to avoid any serious cardiovascular adverse events. As a consequence, only 5 participants completed all treatment conditions, whereas 12 participants completed the placebo and 100 mg 4-FA condition.

Stimulants can cause cardiovascular complications that result from overstimulation of the autonomic nervous system through the release of norepinephrine and dopamine. Adverse cardiovascular events have been reported after recreational use of cocaine and (meth)amphetamine and may include stroke, myocardial infarction, and sudden death (Fischbach, 2017; Lappin et al., 2017). Similarly, sympathomimetic effects such as agitation, tachycardia, hypertension, and hyperthermia have been reported for NPS that are structurally related to stimulants such as mephedrone (Wood et al., 2010; James et al., 2011), MDMA (Turillazzi et al., 2010), MDPV, methoxetamine, and 6-ABP (Hondebrink et al., 2015), sometimes resulting in fatalities (Wood et al., 2010). 4-FA is yet another stimulant that can produce cardiovascular complications that are potentially life-threatening as shown in a series of clinical case reports (Wijers et al., 2017). The present study confirms the clinical suspicions that 4-FA can produce cardiovascular adverse events and that these can already appear at doses that are typically being used in a recreational setting.

The effects of 4-FA on blood pressure and subjective high were dissociable from the time course of 4-FA concentrations in serum. Serum levels of 4-FA peaked at 1–2 h after administration and from there on slowly decreased over time, reaching about 50% of its maximal concentration at 12 h post drug. Subjective rating of high as well as diastolic and systolic blood pressure also peaked at approximately 1–2 h postadministration but declined and returned to baseline levels within 6–8 h. Plotting subjective and autonomic effects against serum concentration resulted in a clockwise hysteresis, suggesting that participants developed acute tolerance to the subjective and pressor effects of 4-FA. At similar serum drug concentrations, subjective and pressor responses were greater on the ascending compared with the descending limb of the concentration-effect curve. Similar manifestations of acute tolerance have been reported after acute administrations of D-amphetamine (Brauer et al., 1996), MDMA (Hysek et al., 2014), and cocaine (Ambre et al., 1988). Alternatively, the clockwise hysteresis loop might have been caused by additional pharmacokinetic or pharmacodynamics factors such as a disequilibrium between arterial and venous concentrations, active antagonistic metabolites, or negative feedback regulation (Louizos et al., 2014).

Participants did not appear to develop acute tolerance for the effects of 4-FA on heart rate. Heart rate temporarily increased between 30 and 60 min post drug but generally remained at baseline levels for about 4 h and began to rise steadily thereafter, at a time when serum levels started to decline. A similar pattern of physiologic response has been previously reported for D-amphetamine and MDMA (Brauer et al., 1996; O’cain et al., 2000; Dolder et al., 2017) and is believed to arise from a cardiovascular baroreceptor reflex function which causes heart rate to decrease at elevated blood pressure (O’cain et al., 2000). Though there were no clear indications of bradycardia following the initial 4-FA induced pressor effect, heart rate only started to systematically increase after blood pressure values returned to baseline. The consequence of such a pattern may be relevant for users who repeatedly self-administer 4-FA to sustain a desired level of euphoria. They may be at an increased risk for cardiotoxicity because subjective feelings of high already dissipate when serum levels of 4-FA and heart rate are still elevated or on the rise.

The effects of 4-FA on mood were most distinct at 1 h post drug and included significant elevations of vigor, friendliness, elation, arousal, and positive mood. Most of these feelings had returned to baseline at 4 h post drug. After this point, feelings of confusion and fatigue increased and the latter even pertained up until 11 h post drug. This seems to indicate that positive or desirable mood changes caused by 4-FA are transient and substituted by less pleasant feelings over time, when serum concentration are decreasing. Mood changes observed after 4-FA are very similar to those that have been reported after single doses of MDMA (Kuypers et al., 2008; van Wel et al., 2012; de Sousa Fernandes Perna et al., 2014) and D-amphetamine (Kelly et al., 2009; Weafer and de Wit, 2013; Kirkpatrick et al., 2016). However, drug-induced negative affect such as fatigue, anxiety, and confusion presented in parallel to increments in positive affect in some of these studies (Kuypers et al., 2008; Kelly et al., 2009). Changes in mood induced by 4-FA thus appear very similar to those observed after MDMA and D-amphetamine, but their order of presenting might differ.

4-Fluoroamphetamine improved tracking performance in the divided attention task and decreased reaction time in the spatial memory task. Improvements in attention and motor performance have been reported for many stimulant drugs such as MDMA, amphetamine, and methylphenidate (Ramaekers et al., 2006; Silber et al., 2006; Bhattacharya et al., 2015; MacQueen et al., 2017). We also observed a significant treatment effect on the number of false alarms in the divided attention task. Here, however, this effect seemed to be driven by a worsening of performance at 4 h after administration in the placebo treatment rather than a change in performance after 4-FA, and may reflect a spurious finding. 4-FA also reduced spatial memory performance as compared to placebo, but this difference just failed to reach statistical significance. Acute memory impairment is well known to occur during MDMA exposure. Several studies have demonstrated that single doses of MDMA produce transient impairment of verbal and spatial memory (Kuypers and Ramaekers, 2005, 2007; Kuypers et al., 2016). The impairing properties of MDMA are primarily related to its serotonergic mechanism of action (Kuypers and Ramaekers, 2005) and can be blocked by 5HT_2_ antagonists (van Wel et al., 2011). 4-FA also increases serotonin levels, although to a lesser degree than MDMA, which opens the possibility that borderline memory impairment observed in the present study potentially represents a clinical phenomenon that may become apparent when tested in larger sample sizes. Overall, however, the current study shows that the neurocognitive profile of 4-FA much resembles enhancement or neutral effects on performance seen with other stimulants.

Participants were also instructed to note adverse effects in a diary during the days following treatment. The number of sub-acute adverse events increased in comparison to those experienced at the actual day of treatment. Sub-acute complaints that were most often reported included headache, fatigue, difficulty concentrating, and anhedonia. Sub-acute mood dips have previously also been reported in MDMA users and are suggested to result from attenuation of 5-HT function for a period following acute use of MDMA (Verheyden et al., 2002; Vizeli and Liechti, 2017). Amphetamine (Chan-Ob et al., 2001) and cocaine (Sofuoglu et al., 2005) abusers have also been reported to experience anhedonia, depression, and fatigue during periods of acute and subacute withdrawal that arises from a state of functional dopamine depletion in the brain during abstinence. The sub-acute effects of 4-FA therefore might be related to depletion of any of these monoamines following excessive suppletion during the intoxication phase. It is noteworthy that sub-acute symptoms already presented in up to 40% of the participants after receiving a single dose of 4-FA. Sub-acute mood dips can be expected to be even more prominent in 4-FA users who use the drug intensively over a prolonged period of time.

In summary, single dose administrations of 4-FA produced significant cardiovascular complications. The drug produced strong elevations in blood pressure during 4 h after administration that were followed by a sustained increase in heart rate. Administration of the high dose of 4-FA was canceled to avoid any serious cardiovascular adverse events. The influences of 4-FA on mood and neurocognition were very similar to those observed with stimulants such as D-amphetamine and MDMA and generally pointed in the direction of enhancement of mood and performance. Negative affect was also reported during the acute phase but more so during the subacute phase. The present data confirm previous clinical observations among 4-FA users and warrant warnings about potential health risks associated with 4-FA use.

## Author Contributions

ET, KK, and JR designed the research study. EdSFP, NH, and NM performed the study. ST performed the pharmacokinetic analyses. PD performed data analyses and produced graphics. EdSFP and JR analyzed the data and wrote the manuscript.

## Conflict of Interest Statement

The authors declare that the research was conducted in the absence of any commercial or financial relationships that could be construed as a potential conflict of interest.
